# Prospective Quantitative and Phenotypic Analysis of Platelet-Derived Extracellular Vesicles and Its Clinical Relevance in Ischemic Stroke Patients

**DOI:** 10.3390/ijms252011219

**Published:** 2024-10-18

**Authors:** Joanna Maciejewska-Renkowska, Justyna Wachowiak, Magdalena Telec, Maria Kamieniarz-Mędrygał, Sławomir Michalak, Radosław Kaźmierski, Wojciech Kociemba, Wojciech P. Kozubski, Maria Łukasik

**Affiliations:** 1 Department of Neurology, Poznan University of Medical Sciences, ul. Przybyszewskiego 49, 60-355 Poznan, Poland; mtelec@ump.edu.pl (M.T.); wkozubski@ump.edu.pl (W.P.K.); 2 Laboratory of Flow Cytometry and Vascular Biology, Department of Neurology, Poznan University of Medical Sciences, 60-355 Poznan, Poland; 3 Division of Neurochemistry and Neuropathology, Department of Neurology, Poznan University of Medical Sciences, 60-355 Poznan, Poland; justynawachowiak@ump.edu.pl (J.W.); swami@ump.edu.pl (S.M.); 4 Department of Neurology, S. T. Dąbrowski Hospital, 62-040 Puszczykowo, Poland; maria.kamieniarz@gmail.com; 5 Department of Neurosurgery and Neurotraumatology, Poznan University of Medical Sciences, 60-355 Poznan, Poland; 6 Department of Neurology, Collegium Medicum, University of Zielona Gora, 65-046 Zielona Gora, Poland; rkazmierski@ump.edu.pl; 7 Department of Radiology, HCP Medical Center, 61-001 Poznan, Poland; wkociemba@wp.pl

**Keywords:** extracellular vesicles, platelets, ischemic stroke, flow cytometry, platelet activation

## Abstract

The levels of platelet-derived extracellular vesicles (pEVs) have been reported as elevated in acute ischemic stroke (IS). However, the results of studies remain equivocal. This prospective, case-control study included 168 patients with IS, 63 matched disease controls (DC), and 21 healthy controls (HC). Total pEVs concentration, the concentration of phosphatidylserine-positive pEVs (PS^+^pEVs), the percentage of PS^+^pEVs (%PS^+^pEVs) and the concentration of pEVs with expression of CD62P^+^, CD40L^+^, CD31^+^, and active form of GPIIb/IIIa receptor (PAC-1^+^) were assessed on days 1, 3, 10, and 90 with the Apogee A50-Micro flow cytometer. The concentrations of pEVs, PS^+^pEVs, and %PS^+^pEVs were significantly higher after IS vs. HC (*p* < 0.001). PS^+^pEVs were higher after stroke vs. controls (*p* < 0.01). The concentrations of pEVs with expression of studied molecules were higher on D1 and D3 after stroke vs. controls. The concentration of pEVs after platelet stimulation with ADP was significantly diminished on D3. IS most notably affects the phenotype of pEVs with a limited effect on the number of pEVs. Ischemic stroke moderately disturbs platelet microvesiculation, most notably in the acute phase, affecting the phenotype of pEVs, with a limited impact on the number of pEVs.

## 1. Introduction

Platelet-derived large extracellular vesicles (pEVs) are a heterogeneous group of blebs formed from the plasma membrane of platelets containing fragments of platelet cytoplasm. Large EVs of platelet origin play an important role in many biological processes, both physiological and pathological, and pEVs demonstrate manifold properties, e.g., procoagulant, proinflammatory, and anticoagulative [1]. Levels of circulating pEVs are described as elevated in various pathological conditions, e.g., diabetes, atherosclerosis, acute coronary syndrome, and ischemic stroke [2,3,4,5]. pEVs express various molecules present on the platelet surface, such as P-selectin (CD62P), GPIIb/IIIa receptor, CD40L, and platelet endothelial cell adhesion molecule-1 (PECAM-1, CD31). P-selectin present on the pEVs surface interacts with monocytes via P-selectin glycoprotein ligand-1 (PSGL-1). It results in the production of proinflammatory cytokines, and exposure of tissue factor (TF) on the monocyte surface [6]. CD40L interacts with CD40-expressing cells such as other platelets, endothelial cells (ECs), lymphocytes, or dendritic cells. Moreover, CD40L binds to the GPIIb/IIIa receptor as strongly as fibrinogen, inducing platelet activation and outside-in signaling, which promotes their aggregation [7]. Interaction of CD40 present on the platelet surface with CD40L causes the release of dense and α granules [8]. pEVs may also express PECAM-1, a key player in the adhesion between ECs and leukocytes. Negatively charged phosphatidylserine present on the pEVs surface promotes coagulation by binding clotting factors and creating a surface for their activity [9].

The number of circulating pEVs is believed to serve as a marker of platelet activation during acute vascular ischemic events [10]. However, in vivo, the process of microvesiculation is complex, and stimulation of different platelet activation pathways does not have to result in the same intensity of pEVs formation. On the other hand, there are equivocal data on the microvesiculation in only partially activated platelets and the role of the GPIIb/IIIa receptor in this process. Since the surface expression of antigens on pEVs reflects the status of platelet activation, the composition of antigens on the pEVs surface may reflect which activation pathways are being promoted. For the above reasons, it seems worthwhile not only to observe the quantitative changes in circulating pEVs, but also to investigate the changes in pEVs phenotype by referring it to the clinical settings in the successive stages of stroke.

To date, there have been very few prospective clinical studies regarding changes in platelet microvesiculation during acute stroke, mostly conducted on small populations, suggesting that pEVs and some of their phenotypes provide new information about the outcome and risk of recurrence [10,11,12,13,14]. Although researchers agree that the level of circulating pEVs in stroke patients is higher than in healthy volunteers, the results of comparisons to controls burdened with vascular disease risk factors are few and inconsistent [10,15]. Thus, our study aims to determine levels of circulating pEVs and their phenotype in the acute and convalescent phases of ischemic stroke compared to a healthy control group (HC) and a control group with vascular disease risk factors (DCs). Moreover, to observe qualitative and quantitative changes in platelet vesiculation in response to stimulation under controlled conditions, we activated ex vivo platelets from patients on days 1, 3, and 10 after ischemic stroke with agonists—ADP or thrombin receptor activating peptide (TRAP) or arachidonic acid (AA)—and compared the results to those obtained in healthy controls.

## 2. Results

### 2.1. Microvesiculation after Stroke

**Figure 1:**
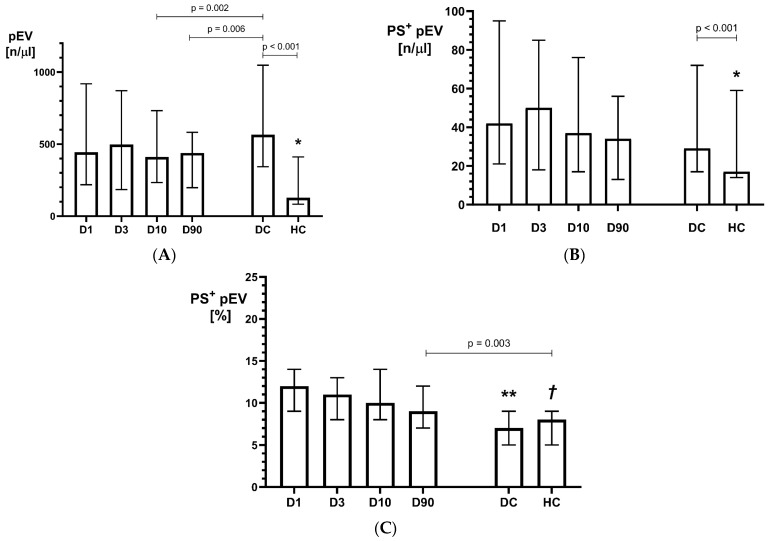
Concentrations of pEVs (**A**), PS^+^pEVs (**B**), and %PS^+^pEVs (**C**) on D1, D3, D10, and D90 of ischemic stroke versus disease controls (DCs) and healthy controls (HCs). * D1-D90 vs. HCs *p* < 0.001; ** D1-D90 vs. DCs *p* < 0.0001; *†* D1-D10 vs. HCs *p* < 0.01. Values are expressed as median (box) and IQR (whiskers).

In acute (D1 and D3), subacute (D10), and convalescent (D90) phases of stroke, the concentration of pEVs (Figure 1A), the concentration of PS^+^pEVs (Figure 1B), and the percentage of PS^+^pEVs in the pEVs population (%PS^+^pEVs, Figure 1C) were significantly higher than in healthy controls (HCs). However, the concentration of pEVs on D1 and D3 did not differ from those observed in the control group with vascular disease risk factors (DCs, disease controls), and it was even lower in stroke subjects than in DCs on D10 and on D90 (Figure 1A). The concentration of PS^+^pEVs on D1-D90 did not differ from those observed in DC; however, %PS^+^pEVs were significantly higher after stroke when compared to both the DCs and HCs (Figure 1C). The highest number of both pEVs and PS^+^pEVs was observed on D3, but the differences in those parameters between studied time points were not significant (Figure 1B). No significant difference in %PS^+^pEVs between the HCs and DCs was found. After stroke, the %PS^+^pEVs gradually decreased over time, and on day 90, the value was still greater than that observed in both control groups (Figure 1C). The concentration of CD62P^+^pEVs, PAC-1^+^pEVs, CD40L^+^pEVs, and CD31^+^pEVs was significantly greater on D1 and D3 as compared to the DCs and significantly greater on D1-D90 than values observed in the HCs.

**Figure 2:**
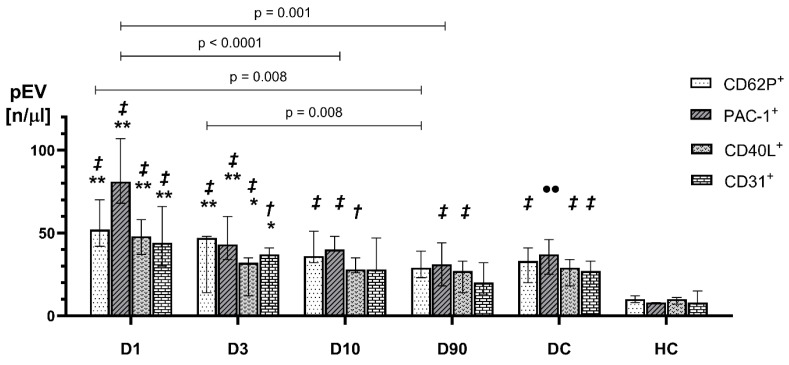
Concentrations of CD62P^+^pEVs, PAC-1^+^pEVs, CD40L^+^pEVs, and CD31^+^pEVs on D1, D3, D10, and D90 of ischemic stroke versus disease controls (DCs) and healthy controls (HCs). ** *p* < 0.001 vs. DCs; * *p* < 0.01 vs. DCs; ***‡*** *p* < 0.01 vs. HCs; ***†*** *p* < 0.05 vs. HCs; •• *p* < 0.001 vs. HCs. Values are expressed as median (box) and IQR (whiskers).

As compared to D1, a significant decrease in the concentration of PAC-1^+^pEVs on D10 and D90 and a significant decrease in the concentration of CD62P^+^pEVs on D90 as compared to D1 and D3 were observed. The concentrations of CD40L^+^pEVs, and CD31^+^pEVs did not change in the studied period and were the same as values observed in the DCs and higher than those in HCs (Figure 2).

### 2.2. Release of pEVs from Non-Stimulated Platelets and Platelets Activated with Agonists in Healthy Donors

Stimulation of platelets with agonists in healthy donors resulted in a significant increase of the concentration of both pEVs and PS^+^pEVs, while the %PS^+^pEVs remained unchanged as compared to the number of pEVs in non-stimulated ex vivo blood samples (Table 1).

After stimulation with ADP, only the concentration of PAC-1^+^pEVs increased. The stimulation with TRAP resulted in significantly increased numbers of CD62P^+^pEVs, CD40L^+^pEVs, and PAC-1^+^pEVs. The result of activation with AA was a significant increase in concentrations of pEVs with expression of every studied molecule (Table 1, Figure S3A–G in Supplementary Material S3).

### 2.3. Release of pEVs from Non-Stimulated Platelets and Platelets Activated with Agonists in Patients after Ischemic Stroke

Day 1. In stroke patients on day 1, the stimulation of platelets with all agonists resulted in a significant increase of the concentration of pEVs, but did not affect the %PS^+^pEVs. A significant increase in the concentration of PS^+^pEVs was observed after activation with TRAP and AA.

Activation with AA resulted in an increased concentration of CD62P^+^pEVs only. Interestingly, activation with ADP resulted in a significantly lower concentration of PAC-1^+^pEV.

Day 3. On day 3, under ADP stimulation, none of the studied parameters changed. The pEVs and PS^+^pEVs increased only under TRAP stimulation. In addition, TRAP as AA increased the concentration of pEVs with the expression of CD62P or PAC-1 or CD40L while AA additionally increased the concentration of CD31^+^pEVs.

Day 10. The effect of AA stimulation on vesiculation resulted in an increased concentration of CD62P^+^pEVs or PAC-1^+^pEVs. On day 10, the platelet stimulation with ADP and TRAP increased the concentrations of all studied pEVs populations, and unexpectedly slightly decreased the %PS^+^pEVs (Table 1, Figure S3A–G in Supplementary Material S3).

### 2.4. Clinical Implications

#### 2.4.1. Stroke Etiology

There were no differences in the concentration of pEVs in general and pEVs with studied phenotypes between patients with different stroke etiology.

**Figure 3:**
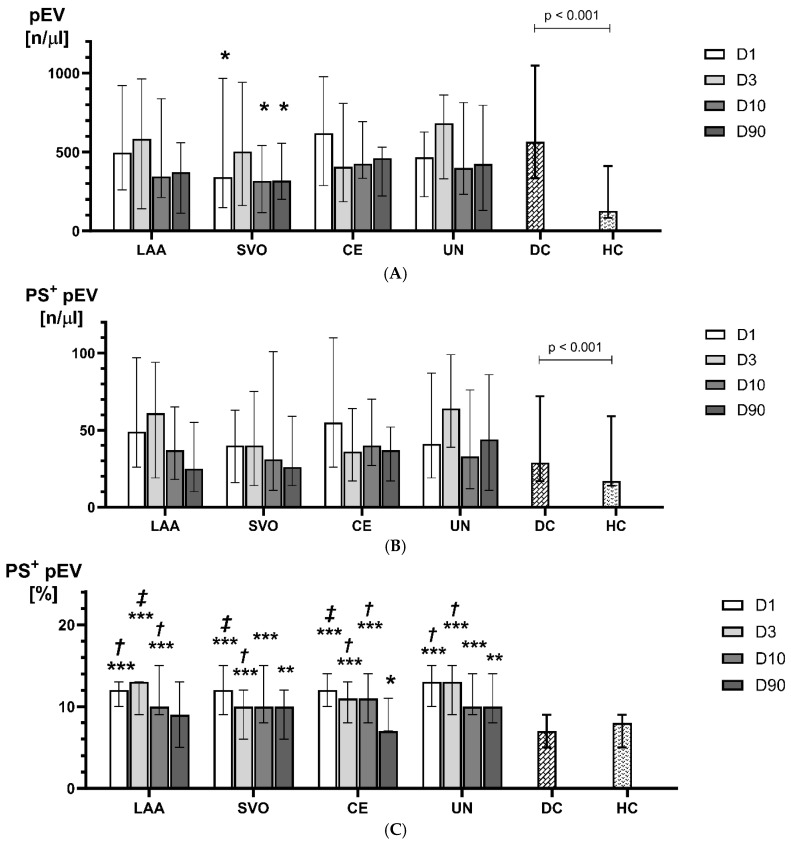
Concentrations of pEVs (**A**), PS^+^pEVs (**B**), and %PS^+^pEVs (**C**) on D1, D3, D10, and D90 of ischemic stroke versus disease controls (DCs) and healthy controls (HCs) depending on the stroke etiology. *** *p* < 0.001 vs. DCs; ** *p* < 0.01 vs. DCs; * *p* < 0.05 vs. DCs; ***‡*** *p* < 0.01 vs. HCs; ***†*** *p* < 0.05 vs. HCs.

A non-significant increase in pEVs concentration was observed on day 3 in patients with stroke of large artery atherosclerosis (LAA), small vessel occlusion (SVO), and undefined etiology while in subjects with cardioembolic etiology (CE), a gradual but non-significant decline was observed from day 1 to day 90 after stroke (Figure 3).

#### 2.4.2. Clinical Outcome, Stroke Lesion Volume, and Treatment

On day 1, a negative correlation was observed between the concentration of PAC-1^+^pEVs and the score on NIHSS (rS = −0.38; *p* < 0.05), and a positive correlation with prognostic SSS (rS = 0.41; *p* < 0.01)—it means the more PAC-1^+^pEVs, the better the clinical status and lesser neurologic deficit on D1. The concentration of circulating PAC-1^+^pEVs on D1 was lower in patients with a poor outcome on day 90 than in those with a good one: 7 (4–21) vs. 10 (5–33), *p* = 0.03.

Nevertheless, in the logistic regression analysis model including other independent and well-recognized risk factors of poor outcome on D90—age, stroke lesion volume on D1 and CRP D1—PAC-1^+^pEVs on neither D1 nor D10 were a significant factor for the clinical status in the convalescent phase of stroke. Moreover, PAC-1^+^pEVs concentration on D3 positively correlated with stroke lesion volume on D1 (rS = 0.46; *p* < 0.01), although this association was not significant in the model of multiple regression analysis including age, systolic blood pressure D1, glycemia D1, and CRP D1.

We also compared the concentration of pEVs in patients treated at admission with intravenous (i.v) thrombolysis with those not treated in this way, and we did not find any differences in studied pEVs parameters. Thus, it seems that i.v. thrombolysis with alteplase in a standard dose of 0.9 mg/kg does not affect the pEVs formation. No significant differences depending on the type of oral anti-thrombotic therapy in studied pEVs parameters at any studied time points were found.

## 3. Discussion

Our study is one of the largest clinical studies on pEVs in stroke, performed in a prospective manner with two different control groups. The results are consistent with the currently prevalent trend that the concentration of pEVs is raised after ischemic stroke when compared to healthy controls; however, regarding controls with vascular disease risk factors (DCs), we found generally opposite tendencies on D10 and D90 and no differences in the acute phase of stroke. It seems that a crucial issue for correct data interpretation is how the pEVs are defined. Chiva-Blanch et al. [10], comparably to our results, found that the concentration of circulating microvesicles did not change significantly during the 90-day observation period after stroke. However, the levels of pEVs described as CD61^+^/AV^+^, CD61^+^/CD142^+^/AV^+^, and CD62P^+^/AV^+^ were elevated after ischemic stroke when compared to the control group with the cardiovascular risk factors. Such defined microvesicles make direct comparisons with our results impossible except for CD61^+^/AV^+^, which correspond with PS^+^pEVs in our study. Nevertheless, we did not find any significant differences in concentration of PS^+^pEVs between stroke patients and DCs. On the other hand, Yao et al. [14] included two different control groups, similarly as in our established protocol. The pEVs defined by flow cytometry as events, both CD41- and lactadherin-positive, were nearly two-fold higher in vascular risk controls and almost three-fold higher in patients after stroke when compared to healthy controls, but lactadherin-negative events were not taken into consideration. PS exposure is known to be related to procoagulant properties of EVs [16], but not all pEVs should be considered as PS^+^. Arraud et al. [17] and Connor et al. [18] reported that, respectively, 50% or 20% of platelet-derived microvesicles in healthy donors bind annexin V. It complies with our previous [15,19] and current findings performed with a dedicated cytometer, indicating that the proportion of %PS^+^pEVs is even lower (9–12% in stroke patients, 7% in DCs, and 8% in HCs). We observed that the percentage of PS^+^pEVs gradually decreased over time, although the differences between studied time points were not statistically significant. Nevertheless, the %PS^+^pEVs was significantly higher at every studied time point after stroke than in DCs or HCs. In the study by Lundström et al. [13], contrary to our findings, most EVs were PS^+^. Moreover, the %PS^+^pEVs seems to be quite a stable parameter since the ex vivo stimulation of platelets did not affect the %PS^+^pEVs. We assumed that the different methodology (i.a. pEVs definitions, devices applied, different reference groups, and periods after stroke) renders the results non-comparable despite the relatively large number of studies in this area.

To validate the applied method and eliminate methodological errors, we assessed microvesiculation using platelet stimulation with agonists that activate different pathways of signal transduction. We performed the assessment in healthy participants (HCs) and in the acute and subacute phase of stroke. Performing platelet stimulation tests in healthy subjects allowed a reference model of platelet microvesiculation to be established in the physiological environment. We assumed that agonists would increase microvesiculation and the expression of molecules on the pEVs surface would be adequate for their increased expression on the surface of activated platelets, in a manner specific to a given agonist. ADP is a weak agonist; it reacts with P2Y_1_ and P2Y_12_ receptors and results in a change of platelet shape, platelet aggregation, and release of dense granules in particular. Further release of ADP from dense granules acts in an autocrine positive-feedback manner and leads to conformational change of the GPIIb/IIIa receptor into its active form (an ‘inside-out’ mechanism), which may be detected by PAC-1 antibody with flow cytometry [20]. In turn, stimulation with TRAP (a synthetic peptide that binds to the PAR1 receptor, the same that thrombin binds) strongly activates platelets and triggers their aggregation and secretion of P-selectin and CD40L from α-granules. Thus, platelet activation ex vivo with TRAP results in increased expression of P-selectin and CD40L on the platelet surface and theoretically on pEVs [21,22].

The results observed in HCs strongly confirmed the expected physiological processes—stimulation of platelets with ADP resulted in enhanced microvesiculation of pEVs, also those with PS or the active form of the GPIIb/IIIa receptor on the surface (PAC-1^+^pEVs), with no significant effect on the number of pEVs with expression of CD62P, CD40L, and CD31. The concentration of PAC-1^+^pEVs increased after stimulation, independently of the used agonist. Stimulation with TRAP, as expected, resulted in an increased concentration of pEVs with surface expression of CD62P or CD40L. Agonists such as ADP and TRAP did not increase the concentration of pEVs with CD31 expression while it was the case after platelet stimulation with AA.

However, in patients in the acute phase of stroke, platelet activation with ADP enhanced microvesiculation only on day 1 (D1), but not on day 3 (D3). Moreover, in contrast to HCs, stimulation of platelets with ADP unexpectedly resulted in a significantly diminished concentration of PAC-1^+^pEVs (D1). According to our knowledge, this is the first clinical evidence that the signaling via P2Y_1_ and P2Y_12_, engaging the GPIIb/IIIa receptor in an inside-out mechanism, is involved in the release of pEVs. It might mean that in acute stroke, the platelet activation pathway via purinergic receptors, physiologically resulting in microvesiculation, is significantly disturbed, while the pathway via PAR1 (activated with TRAP) is affected only on D1 (Table 1). One cannot exclude that limited microvesiculation under ADP on D3 might be associated with in vivo overactivation and exhaustion of the ADP-purinergic pathway on D1. It may be accompanied by a diminished concentration of PAC-1^+^pEVs, apparently as a consequence of diminished platelet surface expression of GPIIb/IIIa in its active form on D1 (Table 1). It may be supported by the fact that treatment with inhibitors of both GPIIb/IIIa and purinergic receptors reduced the concentration of pEVs while ASA does not show such properties [23]. On the other hand, as suggested by McCabe et al. [24], decreased binding of PAC-1 by platelets after stroke might be the result of a blockage of binding sites by fibrinogen, and that with fewer receptors occupied by the natural ligand, less aggregation occurs. However, we observed in our previous study [25] that reduced binding of PAC-1 by platelets was accompanied by lowered fractions of homo- and heterologous platelet aggregates. It suggests diminished surface expression of the GPIIb/IIIa receptor and disturbed GPIIb/IIIa inside-out signaling during the acute phase of stroke rather than intensive receptor binding with the ligand. This limited release of pEVs expressing active GPIIb/IIIa under ADP stimulation may be protective, since we observed on D1 an association between a lower concentration of PAC-1^+^pEVs and better clinical outcomes on both D1 and D90. Nevertheless, the concentration of PAC-1^+^pEVs seems not to be such a strong determinant, since the correlation of PAC-1^+^pEVs with the outcome became statistically non-significant after including in the model other factors affecting neurological status. Moreover, in stroke subjects on D1, increased microvesiculation under TRAP stimulation was present. However, unexpectedly, this did not translate into an increased concentration of pEVs with surface expression of the studied molecules. Thus, on D1, the activation of platelets with ADP or TRAP resulted in increased pEVs release with significantly limited transformation of GPIIb/IIIa to the active form or limited degranulation of α-granules. It might confirm previous observations that pEVs release does not have to occur in parallel with other processes resulting from platelet activation [25,26,27,28].

Arachidonic acid, is used ex vivo to activate platelets via the thromboxane receptor (TP). This process may be inhibited by acetylsalicylic acid (ASA). In healthy controls, AA enhances platelet microvesiculation, but this effect is less prominent in stroke patients on D1 and vanishes on D3 and D10. We suppose that it may be a consequence of ASA treatment after ischemic stroke, although it is currently thought that the in vivo effect of ASA on circulating pEVs is very limited [19,23,29]. To conclude, we consider the reduced platelet microvesiculation after ex vivo platelet stimulation in ischemic stroke patients as an unexpected result.

The present study did not detect the previously reported quantitative differences in pEVs depending on stroke etiology. However, it was observed that only in patients with stroke of cardioembolic etiology (CE), the concentration of pEVs was the highest on the first day and then decreased, while in other patients, the concentration of pEVs on D3 was higher than on D1. Nevertheless, these differences were not significant, and the hopes that dynamics of pEVs vesiculation might be considered markers of CE were not fulfilled. In clinical practice, unambiguous confirmation of CE is challenging, especially as cardioembolism implies different stroke preventive options. We did not observe any differences in pEVs parameters depending on the type of anti-thrombotic treatment. Based on numerous previous studies, the influence of treatment with ASA on microvesiculation in vivo seems to be negligible, while the effect of treatment with anticoagulants in humans is not well known and remains equivocal [19,23,30,31]. An unquestionable depressive effect on the microvesiculation is caused by P2Y12 inhibitors, not used in this study [32,33].

Although this study is based on a relatively large population, the differences between patients and controls burdened with vascular disease risk factors were not prominent and pEVs are not able to clearly discriminate between the groups. This phenomenon can be interpreted in different ways. Firstly, ischemic stroke may not affect vesiculation as significantly as was previously thought. Secondly, the primarily enhanced microvesiculation is diminished by the binding of pEVs to the microvasculature or, thirdly, by intense pEVs turnover with elimination by increased phagocytosis or endocytosis. The disease control group was matched to the patient group according to the vascular disease risk factors. Therefore, the lack of differences in the acute phase of stroke may suggest that enhanced microvesiculation is a chronic process. It determines neither the cause nor the result of ischemia and probably results from the chronic nature of the vascular disease and stroke risk factors, not the ischemia itself. We suppose that the phenotypic changes of pEVs in the acute phase, which gradually wane with time, may play a more significant role in this setting. Thus, exploring still undiscovered nuances of phenotypic analysis of pEVs more than pEV levels seems reasonable, especially in the context of dynamic changes of the phenotype of pEV subpopulations during the stroke. The translational study by Lundstrom et al. [13] detected a positive association between the tissue factor-bearing pEVs subpopulation defined as PS^-^TF^+^PMV and primary outcome (recurrent ischemic stroke and acute myocardial infarction). Moreover, our previous study [34] revealed elevated levels of CD62P+/CD61+, PAC-1+/CD61+, and CD31+/CD61+ pEVs in patients with recurrent vascular events during a one-year follow-up. Nevertheless, it seems that wider diagnostic application of this method at the patient’s bedside would be premature. Nevertheless, it seems that wider diagnostic application of this method at the patient’s bedside would be premature.

Despite the significant growth of the extracellular vesicles research field, a unified protocol of flow cytometry extracellular vesicles evaluation is still lacking and a direct comparison of results is still unfeasible. The results of a comparative study by Berckmans et al. [35] documented immense inconsistency in the quantitative estimation of EVs between two different protocols established in a 17-year interval.

The major study limitation is the use of only one method of pEVs assessment. Nevertheless, we made attempts to mitigate this limitation. Since only a few studies have implemented two different control groups, we at least partly filled the gap in the field including both DCs and HCs. Moreover, nanoparticle flow cytometry makes it possible to evaluate pEVs both quantitatively and qualitatively. Our study is one of the largest prospective studies on pEVs in stroke according to our knowledge, and to validate the used method we assessed microvesiculation under controlled conditions. It provided better insight into the activation pathways involved in pEVs release, both in stroke patients and in healthy donors.

## 4. Materials and Methods

### 4.1. Studied Populations

Patients’ characteristics are shown in Table 2 and Table 3. A total of 1279 consecutive patients who presented with suspected stroke in the Stroke Unit at the Department of Neurology and Cerebrovascular Disorders of Poznan University of Medical Sciences, at L. Bierkowski Hospital, Poznan, Poland between January 2017 and May 2019 were screened for inclusion in the study. Inclusion criteria were as follows: ischemic stroke confirmed based on clinical and radiological evidence in cranial computed tomography (CT) scans and/or brain MRI at admission, age over 45, and symptoms onset within 24 h prior to clinical evaluation and blood sampling. The exclusion criteria are summarized in Figure S1 in Supplementary Material S1. The summary of treatment received by patients at admission and during the study is available in Table S4 in Supplementary Material S4. Finally, 168 patients were included in the study.

Clinical assessment was performed in the acute phase of stroke, on days 1 (D1, *n* = 168) and 3 (D3, *n* = 167), in the subacute phase on day 10 or at discharge (D10, *n* = 163) and on day 90 ± 3 days (D90, *n* = 93) after stroke (convalescent phase). It included physical and neurological examination and the subjects were assessed on the National Institutes of Health Stroke Scale (NIHSS), Scandinavian Stroke Scale (SSS), and modified Rankin Scale (mRS). The outcome was assessed on D10 and D90 and it was defined as poor when the score on the mRS was ≥3. The etiology of the stroke was classified according to the TOAST classification [36]. The standard diagnostic measures included blood pressure and height and weight assessments to calculate body mass index (BMI). The clinical evaluation was supplemented by laboratory investigations including blood count with automatic smear test, coagulation, biochemical and urine tests, and electrocardiography (ECG), chest radiograph, color Doppler duplex ultrasonography, transcranial Doppler ultrasonography (TCD), and transthoracic +/− transoesophageal echocardiography. The MRI scans were performed twice: within 24 h from the symptom onset and 90 ± 3 days after stroke. The protocol of the measurement of the infarct volume was described previously [37].

The control group with risk of vascular disease (DCs—disease controls) consisted of 63 patients recruited from the basic healthcare department, who fulfilled inclusion criteria: age over 45 years, no previous acute vascular events in patient history (ischemic stroke, acute myocardial infarction, peripheral arterial occlusion), and presented at least two vascular risk factors: arterial hypertension, hypercholesterolemia, type 2 diabetes, BMI > 30 (kg/m^2^), or smoking.

A total of 21 healthy subjects (HCs—healthy controls) were recruited for this study from the University of the Third Age, the hospital staff, and relatives. Subjects exhibited no vascular risk factors and no history of any vascular incident. Other exclusion criteria included malignancies, autoimmune diseases, inflammatory and hematological disorders, infection, liver and renal failure, alcohol and/or drug abuse. The standard diagnostic measures and routine laboratory investigations were supplemented in the clinical evaluations (Table 2).

The study protocol was designed in accordance with the Declaration of Helsinki, and it was approved by the bioethics committee of Poznan University of Medical Sciences (approval no. 112/2016). Informed consent was obtained from all subjects involved in the study.

### 4.2. Labeling of pEVs and Flow Cytometry Analysis

#### 4.2.1. Blood Samples

Blood samples were collected in the morning hours, after overnight fasting with an 18-gauge needle from the antecubital vein without venostasis. Samples intended for flow cytometry analysis were drawn into a 4.5 mL tube with 0.105 M buffered sodium citrate anticoagulant (Becton Dickinson, Plymouth, UK) and gently reversed right after blood collection to avoid clotting. Citrated whole blood was centrifuged at 1500× *g* for 20 min at room temperature (RT) to obtain platelet-poor plasma (PPP), then plasma was transferred to Eppendorf tubes (Eppendorf, Hamburg, Germany) and centrifuged at 13,000× *g* for 2 min to obtain platelet-free plasma (PFP). The PFP was aliquoted as 250 μL samples, snap-frozen in liquid nitrogen, and stored at −80 °C for further analysis. Immediately after, additional blood was drawn into a 10 mL tube (Sarstedt Monovette, Nümbrecht, Germany) to prepare serum for biochemical tests. Blood samples were coded and blinded for laboratory technicians. All patients’ samples were processed in the same manner, in the time period no longer than 30 min after blood collection (Supplementary Material S2).

#### 4.2.2. Preparation and Labeling of Platelet-Derived Extracellular Vesicles

PFP samples were thawed at RT and then extracellular vesicles (EVs) were isolated from PFP. From a 250 μL PFP sample, after two-step high-speed centrifugation for 30 min at 18,890× *g* and washing twice with a 0.22 μm filtered citrate-phosphate-buffered saline solution (PBS), 100 μL of isolated EVs specimen was obtained. EVs suspension in a volume of 7.5 μL was incubated for 30 min in the dark, at RT with 15 μL of 25 mM CaCl_2_ and antibodies conjugated with fluorochromes in accordance with the three-color cytometry protocol. An amount of 3.75 μL of the antibodies (Ab) phycoerythrin (PE)-conjugated anti-CD61 (platelet-gating Ab, Becton Dickinson, Franklin Lakes, NJ, USA) and allophycocyanin (APC)-conjugated Annexin V (Ab against external phosphatidylserine (PS), BioLegend, San Diego, CA, USA) were used as a platelet-derived extracellular vesicle marker. Fluorescein isothiocyanate (FITC)-conjugated PAC-1 (Ab against active form of GPIIb/IIIa receptor, BioLegend), FITC-conjugated CD62P (Ab against P selectin, BioLegend), CD154/FITC (Ab against CD40L, BioLegend), or CD31/FITC (Ab against PECAM-1, BioLegend) in a volume of 3.75 μL were used independently to receive four separated measurements from one EVs sample. After incubation, all the specimens were diluted with 450 μL of 0.22 μm filtered 0.9% NaCl to finish the incubation before flow cytometry analysis (Supplementary Material S2).

#### 4.2.3. Flow Cytometric Analysis

Analyses of samples were performed with the Apogee A-50 Micro FC (Apogee Flow Systems, Hemel Hempstead, UK) using 488 nm (blue) and 638 nm (red) lasers dedicated to extracellular vesicle detection. ApogeeMix #1493 (Apogee Flow Systems) was used each day of measurements to check FC performance. The pEVs concentration (number of pEVs per μL of prepared purified EVs) was measured automatically based on measured flow rate, sample volume, and the number of fluorescence-positive events (*n*). Platelet-derived EVs were gated using medium-angle light scatter (MALS) and a fluorescence channel and were defined as CD61-positive events in a size over the detection threshold of the Apogee A-50 Micro cytometer (Apogee Flow Systems), i.e., of more than 100 nm (large extracellular vesicles) in color fluorescence plots after staining with CD61-PE antibodies. Phenotypes of circulating pEVs were defined on reactivity to monoclonal antibodies specified above. The gating strategy was presented in a previously published article [15]. We also performed analyses of EVs isolated from the whole blood, unstimulated, and stimulated with platelet agonists to ensure that analyzed dot plots were reliable and represented signals coming from platelet-derived vesicles. We measured the pEVs concentration as the total number CD61^+^pEVs in the sample volume (pEVs/µL), the concentration of pEVs expressing external phosphatidylserine (PS^+^pEVs), the percentage of PS^+^pEVs within the pEVs population (%PS^+^pEVs), and the concentration of pEVs with surface expression of CD62P (CD62P^+^) or PAC-1 (PAC-1^+^) or CD40L (CD40L^+^) or CD31 (CD31^+^pEV). All relevant details about established flow cytometry protocol and analysis are available in the Tables S1–S3 and Figure S2 in Supplementary Material S2.

#### 4.2.4. Analysis of Large EVs Derived from Platelets Stimulated with ADP, TRAP, and Arachidonic Acid in Stroke Patients and Healthy Controls

The stroke patients were randomly selected on D1 for the stimulation tests from the patients included in the study. Platelet activation with agonists was performed in 33 patients on D1, 31 patients on D3, and 28 patients on D10 after stroke and once in 21 healthy subjects. The healthy participants were included to discover a standard pattern of platelet microvesiculation in physiological state and form a reference model. Blood samples were collected according to the protocol described above. The portions of citrated blood in the amount of 1.5 mL were incubated in Eppendorf tubes (Eppendorf) with platelet agonists—7.5 μL 10 mM ADP, 30 μL 0.5 mM TRAP, and 20 μL Aspi, which contains arachidonic acid in a concentration of 0.5 mM—and without any agonist as a reference, at RT, for 15 min. After incubation, the EVs were isolated, labeled, and analyzed following the established protocol described above (Supplementary Material S2).

### 4.3. Statistical Analysis

The sample size was a priori evaluated using standard statistical criteria for the estimation of sample size and statistical power. As we expected to reveal at least 30% differences between the groups with a significance of at least 1% and statistical power of at least 90%, taking into account that the natural variability is at the most 30% and the ratio of case to control sample size equals 1, the estimated minimum sample size was 32 experimental subjects and 32 matched controls. The Shapiro–Wilk test was used to verify the normal distribution of data. Quantitative parameters on the interval scale were expressed as mean ± standard deviation (normal data distribution) and data with distribution other than normal as median with interquartile range (IQR). Qualitative data expressed on ordinal and nominal scales were presented as the number of characteristics with percentage notation (%). Examined pEVs parameters had a distribution other than normal. The significance of intergroup differences was evaluated using the nonparametric Mann–Whitney U test and the Kruskal–Wallis ANOVA test followed by Bonferroni’s correction method. The Wilcoxon test was performed when two dependent groups were compared. Multiple comparisons between dependent variables were examined with a nonparametric Wilcoxon signed-rank test with Bonferroni’s correction. Categorical variables on a nominal scale (2 × 2 tables) were analyzed with the chi-square test or Fisher’s exact test. The *p* value established as statistically significant for two-group comparisons was *p* < 0.05. All data were analyzed using Statistica software v.13.1 (2017) (TIBCO Software Inc., Santa Clara, CA, USA), and GraphPad Prism v. 10.3.0 (GraphPad Software, San Diego, CA, USA).

## 5. Conclusions

When compared to disease controls, ischemic stroke most notably affected the phenotype of pEVs, with a limited effect on the number of circulating pEVs. The concentrations of circulating pEVs in the acute phase of stroke did not differ from those found in the patients burdened with vascular disease risk factors. Thus, pEVs are not able to clearly discriminate between the two groups and more comprehensive diagnostic application of pEVs at the patient’s bedside ought to be postponed. The more particular approach in phenotypic analysis of pEVs subpopulations may be a direction which is worth considering.

The %PS^+^pEVs in the studied populations was relatively low at 7–12%. The %PS^+^pEVs was greater in stroke subjects than in controls (DCs and HCs). The concentrations of pEVs and PS^+^pEVs were higher in stroke subjects than in HCs.

On D1 of ischemic stroke, pEV levels were increased after ex vivo platelet stimulation with ADP, but on D3, the vesiculation of pEVs was disturbed as compared to HCs.

Thrombolytic treatment did not affect pEVs concentration. Since the differences between pEV parameters in different stroke etiologies and between sufferers and subjects at risk of vascular disease are not so prominent, the usefulness of these measurements in clinical practice currently seems to be questionable. Moreover, despite significant correlations in Spearman rank analysis, the concentration of pEVs in multiple regression models ceases to be an independent factor for clinical outcome or ischemic lesion volume.

## Figures and Tables

**Table 1:** Concentration of pEVs and PS^+^ pEVs, percentage of PS^+^ pEVs and concentration of pEVs with surface expression of CD62P, PAC-1, CD40L, and CD31 in healthy controls and patients with ischemic stroke (D1, D3, D10) before (pEV 0) and after platelet stimulation with ADP, TRAP, and AA.

	pEV 0	+ADP	p pEV 0 vs. +ADP	+TRAP	p pEV 0 vs. +TRAP	+AA	p pEV 0 vs. +AA
HCs *n* = 21							
pEVs [n/μL]	128 (83–411)	440 (262–4853)	<0.0001	457 (371–1939)	<0.0001	1354 (68–2639)	<0.0001
PS^+^ pEVs [n/μL]	9 (7–24)	20 (13–274)	<0.0001	26 (20–96)	<0.0001	123 (7–240)	<0.0001
%PS^+^ pEVs [%]	8 (5–9)	5 (3–6)	ns	5 (4–7)	ns	7 (7–9)	ns
pEVs CD62P^+^ [n/μL]	10 (8–12)	9 (8–11)	ns	14 (10–15)	0.004	26 (13–38)	0.005
pEVs PAC-1^+^ [n/μL]	8 (7–8)	15 (11–26)	<0.0001	12 (9–13)	0.0007	15 (15–16)	0.005
pEVs CD40L^+^ [n/μL]	10 (7–11)	9 (6–13)	ns	13 (12–38)	0.0007	10 (6–13)	0.04
pEVs CD31^+^ [n/μL]	8 (7–15)	9 (8–52)	ns	11 (6–12)	ns	16 (15–17)	0.005
D1 *n* = 33							
pEVs [n/μL]	344 (225–416)	1113 (900–1708)	0.0007	574 (485–1993)	0.0005	475 (395–488)	0.001
PS^+^ pEVs [n/μL]	22 (17–86)	26 (25–34)	ns	38 (24–93)	0.0006	29 (27–31)	0.0007
%PS^+^ pEVs [%]	13 (10–15)	12 (10–16)	ns	14 (11–17)	ns	16 (14–17)	ns
pEVs CD62P^+^ [n/μL]	50 (40–66)	39 (36–68)	ns	38 (18–102)	ns	68 (63–74)	0.004
pEVs PAC-1^+^ [n/μL]	77 (65–101)	64 (44–65)	0.002	43 (36–104)	ns	82 (65–100)	ns
pEVs CD40L^+^ [n/μL]	48 (32–54)	40 (35–43)	ns	40 (24–76)	ns	49 (37–50)	ns
pEVs CD31^+^ [n/μL]	39 (33–50)	33 (32–44)	ns	38 (32–62)	ns	44 (22–46)	ns
D3 *n* = 31							
pEVs [n/μL]	326 (316–358)	537 (256–621)	ns	701 (431–962)	0.009	245 (225–680)	ns
PS^+^ pEVs [n/μL]	17 (9–73)	30 (8–47)	ns	25 (18–54)	0.003	14 (12–38)	ns
%PS^+^ pEVs [%]	11 (8–13)	7 (5–12)	ns	14 (12–14)	ns	15 (12–16)	ns
pEVs CD62P^+^ [n/μL]	45 (12–51)	60 (35–67)	ns	70 (45–79)	0.007	52 (47–83)	0.0007
pEVs PAC-1^+^ [n/μL]	40 (32–63)	62 (51–110)	ns	54 (46–68)	<0.0001	62 (44–90)	0.004
pEVs CD40L^+^ [n/μL]	35 (11–42)	36 (35–51)	ns	64 (44–67)	<0.0001	44 (17–66)	0.001
pEVs CD31^+^ [n/μL]	38 (12–44)	29 (27–52)	ns	39 (22–62)	ns	46 (22–69)	<0.0001
D10 *n* = 28							
pEVs [n/μL]	490 (322–742)	1311 (641–2367)	0.0001	1048 (540–1051)	<0.0001	488 (307–491)	ns
PS^+^ pEVs [n/μL]	41 (19–69)	45 (30–59)	0.01	45 (42–54)	0.04	31 (29–33)	ns
%PS^+^ pEVs [%]	10 (8–17)	6 (5–8)	0.04	7 (7–9)	0.04	8 (6–9)	ns
pEVs CD62P^+^ [n/μL]	37 (29–46)	68 (54–84)	<0.0001	64 (55–73)	0.0002	64 (57–84)	0.0002
pEVs PAC-1^+^ [n/μL]	40 (37–44)	76 (72–80)	0.0001	68 (63–100)	0.0002	71 (53–95)	<0.0001
pEVs CD40L^+^ [n/μL]	29 (25–47)	64 (52–74)	0.0002	41 (35–46)	0.0002	40 (28–55)	ns
pEVs CD31^+^ [n/μL]	30 (27–45)	55 (52–70)	<0.0001	61 (50–66)	0.0006	45 (40–61)	ns

ns—*p*-value not statistically significant.

**Table 2:** Baseline characteristics of studied groups.

	Ischemic Stroke Patients D1 N = 168	Control Group with Vascular Disease Risk Factors (DC) N = 63	Healthy Control Group (HC) N = 21	*p* Value Stroke D1 vs. DC
Age, years	69 ± 12	67 ± 13	44 ± 10	0.87
BMI, kg/m^2^	25.4 (23.6-28.4)	27.0 (24.9–30.6)	20.3 ± 0.8	0.76
Female sex, *n* (%)	76 (48.1%)	27 (42.2%)	9 (42.8%)	0.74
Total cholesterol, mM/L (D1)	8.2 ± 1.4	9.8 ± 1.7	4.8 ± 0.3	0.39
Triglycerides, mM/L (D1)	8.8 ± 1.7	9.6 ± 2.1	1.5 ± 0.2	0.12
Glucose, mM/L (D1)	7.2 ±2.6	6.7 ± 1.8	4.6 ± 0.9	0.09
Platelets, T/µL (D1)	236 ± 73	245 ± 67	210 ± 21	0.13
SBP, mmHg (D1)	143 ± 21	137 ± 19	123 ± 14	0.11
DBP, mmHg (D1)	83 ± 11	80 ± 10	71 ± 8	0.07
Arterial hypertension, *n* (%)	126 (75.8)	47 (74.6)	-	0.95
Diabetes mellitus, *n* (%)	38 (22.6)	12 (19.0)	-	0.56
Coronary heart disease, *n* (%)	54 (32.1)	26 (41.2)	-	0.14
Atrial fibrillation, *n* (%)	56 (33.5)	23 (36.5)	-	0.65
Dyslipidemia, *n* (%)	55 (32.5)	22 (34.9)	-	0.76
Smoking, *n* (%)	40 (23.8)	14 (22.2)	-	0.80
Previous stroke, *n* (%)	31 (18.3)	-	-	-
Thrombolysis, *n* (%)	54 (32.1)	-	-	-
ASA, *n* (%)	166 (98.8)	25 (39.7)	-	<0.01
ACE-I, *n* (%)	53 (31.5)	22 (30.1)	-	0.32
Diuretics, *n* (%)	40 (23.8)	19 (39.7)	-	0.08
β-blockers, *n* (%)	63 (37.5)	16 (25.4)	-	0.13
Ca-channel blockers, *n* (%)	28 (16.6)	16 (37.2)	-	0.89
ARB, *n* (%)	17 (10.1)	6 (9.5)	-	0.89
Statins, *n* (%)	161 (95.8)	57 (90.4)	-	0.11
Oral hypoglycemic, *n* (%)	27 (16.1)	7 (11.1)	-	0.34
Insulin, *n* (%)	12 (7.1)	7 (11.1)	-	0.33
Oral anticoagulants, *n* (%)	2 (1.2)	23 (36.5)	-	<0.001

D1—day 1 of stroke; BMI, body mass index; SBP, systolic blood pressure; DBP, diastolic blood pressure; ASA, acetylsalicylic acid; ACE-I, angiotensin-converting enzyme inhibitors; ARB, angiotensin-receptor blockers.

**Table 3:** Clinical characteristics of stroke patients.

Stroke Etiology (TOAST Classification),	n (%)
LAA	58 (34.5%)
SVD	43 (25.6%)
CE	50 (29.8%)
OE	0
UE	17 (10.1%)
Stroke lesion volume,	mL, median (IQR)
Day 1	2.4 (0.5–11.5)
Day 90	1.0 (0.1–6.0)
NIHSS	score, median (IQR)
NIHSS D1	4 (2–8)
NIHSS D3	3 (1–9)
NIHSS D10	2 (1–6)
NIHSS D90	1 (0–4)
mRS D3	1 (1–4)
mRS D10	1 (1–4)
mRS D90	0 (0–1)

LAA, large artery atherosclerosis; SVD, small vessel disease; CE, cardioembolic stroke; OE, stroke of other etiology; UE, stroke of unknown etiology; NIHSS, National Institutes of Health Stroke Scale; mRS, modified Rankin scale.

## Data Availability

The raw data supporting the conclusions of this article will be made available by the authors on request.

## Supplementary Materials

The following supporting information can be downloaded at: https://www.mdpi.com/article/10.3390/ijms252011219/s1. References [38,39] are cited in the Supplementary Materials.
