# Supplementary material for: Prospective Quantitative and Phenotypic Analysis of Platelet-Derived Extracellular Vesicles and Its Clinical Relevance in Ischemic Stroke Patients
Source: Int J Mol Sci. 2024 Oct 18;25(20):11219. doi: 10.3390/ijms252011219 (PMC11508277; doi:10.3390/ijms252011219)
Supplement: Supplementary file 1 [file ijms-25-11219-s001.zip › SM2.pdf]

# Supplementary Material S2

# MIFlowCyt-EV of study "Prospective quantitative and phenotypic analysis of platelet-derived large extracellular vesicles and its clinical relevance in ischemic stroke patients"

## 1 Preanalytical variables and experiment design

### 1.1 Experimental design

The purpose of the flow cytometry measurements (A50-Micro, Apogee Flow Systems, Hemel Hempstead, UK) was to compare the concentrations of extracellular vesicles (EVs) released from platelets ($CD61^+$), the procoagulant EVs amongst platelet-derived EVs ($CD61^+/PS^+$), subpopulations of platelet-derived EVs expressing CD62P ($CD61^+/CD62P^+$), active form of GPIIb/IIIa receptor ($CD61^+$/PAC-$1^+$), CD40L ($CD61^+/CD154^+$) and CD31 ($CD61^+/CD31^+$) in purified suspension made from platelet-free plasma (PFP) between patients after ischemic stroke (IS), healthy controls and patients burdened with risk factors of cardiovascular diseases. Flow cytometry evaluation in patients after IS was performed at four time points – 1, 3, 10, and 90 days after stroke onset – and in healthy controls and patients with cardiovascular risk was performed at three time points: 1, 3, and 10 days after study enrollment. We also performed platelet stimulation tests in citrated blood taken from patients at three time points: 1, 3, and 10 days after ischemic stroke, and in healthy controls. From the blood containing platelets stimulated with three agonists of platelet receptors, we isolated the corresponding EV suspension and analyzed it with the same flow cytometry protocol.

The study involved two flow cytometer operators, and FC analyses were performed between January 2017 and May 2019. The evaluation of samples obtained during platelet stimulation tests took place between April 2019 and June 2020.

### 1.2 Sample preparation – flow cytometry

Blood samples were collected and processed following the guidelines of EV research. (1) Samples were collected in the morning hours, with an 18-gauge needle from the antecubital vein without venostasis, to exclude accidental platelet activation, aggregation, or hemolysis. First, 2 ml of blood were discarded and then samples intended for analysis were collected into two 4.5 ml tubes with 0.105 M (3.2%) buffered sodium citrate anticoagulant (Becton Dickinson, Plymouth, UK). Tubes were gently reversed 3 to 4 times immediately after collection to mix blood with anticoagulant thoroughly. All patients' samples were processed identically during the 30-minute interval after blood collection.

Citrated whole blood was centrifuged at 1500 g for 20 min at room temperature (Eppendorf 5804 R Centrifuge with F-35-48-17 rotor, Eppendorf) to obtain platelet-poor plasma (PPP). Obtained citrated plasma was carefully transferred to 1.5 ml Eppendorf 3810X tubes (Eppendorf AG, Germany), pipetting the plasma 1 cm above the cellular pellet, and centrifuged at 13 000 *g* for 2 min (Eppendorf 5415 D Centrifuge with F-45-24-11 rotor, Eppendorf) to obtain platelet-free plasma (PFP). Then prepared PFP (supernatant), pipetting

the plasma 0.5 cm above the pellet, was aliquoted as 250 µl samples, snap-frozen in liquid nitrogen and stored at −80 °C for further analysis.

Before EV isolation, PFP samples were thawed at room temperature. The first step of EV isolation included high-speed centrifugation of PFP for 30 min at 18890 g (Eppendorf 5804 R Centrifuge with F-45-30-11 rotor, Eppendorf). After centrifugation, 225 µl of supernatant was discarded and 225 µl of 0.22-µm-filtered (0.22 µm Syringe-driven Filter Unit, Millex, Ireland) citrate-phosphate-buffered saline solution (PBS) containing 1.4 mmol/l phosphate, 154 mmol/l NaCl, and 10.9 mM trisodium citrate with pH 7.4 was added to the pellet and then the second centrifugation was performed. After the second step, 225 µl of supernatant was discarded and 75 µl of PBS was added to the pellet, and the sample was briefly mixed on a vortex mixer. Immediately after, samples were placed in liquid nitrogen for 20 min. After this stage, samples were stored at −80 °C. Finally, from a 250 µl PFP sample, 100 µl of isolated EV suspension was obtained.

## 1.3. Sample preparation – platelet stimulation tests

Platelet stimulation tests were performed in blood taken from patients after ischemic stroke at D1 (N=33), D3 (N=31), and D10 (N=28) after stroke onset and from healthy controls (N=21) in a manner described above. From the citrated blood, four identical 1.5 ml portions were obtained and transferred into plastic Eppendorf tubes. The first one, named “0”, represents the platelets in their intact, non-activated state. The remaining three portions of blood were mixed with 7.5 µl of adenosine diphosphate (ADP) in a concentration of 0.5 mM (Sigma-Aldrich), 30 µl of thrombin receptor activator peptide (TRAP) in a concentration of 10 mM (Sigma-Aldrich), and 20 µl of arachidonic acid in a concentration of 0.5 mM (Aspi Test, Roche), respectively. All the samples were incubated for 15 min at room temperature. After incubation, EVs were isolated in a manner described in Section 1.2.

## 1.4 EV staining

EVs were stained with antibodies and Annexin V. The summary of antibodies and reagents used in our protocol is available in Table S2. Each 7.5 µl of isolated EV sample was double-labeled with 3.75 µl of CD61-PE (phycoerythrin) and 3.75 µl of Annexin V-APC (allophycocyanin), and additionally single labeled with one of the antibodies against molecules: with 3.75 µl of CD62P-FITC (fluorescein isothiocyanate), with 3.75 µl of PAC-1-FITC, with 3.75 µl of CD154-FITC, and with 3.75 µl of CD31-FITC. Our established approach assumes using four Eppendorf tubes for the staining procedure and, as a result, producing four independent measurements for one EV sample. Intra-sample and inter-sample variations were determined, and the Pearson's variability coefficients (CV) are 2.3% and 5.3% for the percentages of CD61- and AnV-positive EV, respectively, and 3.2% and 7.3% for the $CD62P^+$, $PAC\text{-}1^+$, $CD40L^+$, and $CD31^+$ events. Since the coefficients of variation was low, the final value of measurement was the mean value of four measurements.

Calcium chloride solution in a concentration of 25 mM, pre-filtered with a 0.22 µm-diameter filter, was added to each tube in the amount of 15 µl and samples were incubated for 30 min in the dark, at room temperature. Then, 450 µl of 0.9% NaCl was added to each tube to stop the reaction.

It is known that sodium citrate acts as an anticoagulant by binding $Ca^{2+}$ to prevent the blood from clotting. As a result, the citrated plasma and, further, isolated EVs, are calcium-depleted. It seems to be reasonable to recalcify the EV environment during staining with Annexin V, because Annexin V may require an appropriate $Ca^{2+}$ concentration to achieve optimal conditions for its binding with phosphatidylserine on the surface of EVs. (2)

## 1.5 Buffer-only control

Each day of work with the flow cytometer started with cleaning the tube system with 7.5-10% NaClO solution at least once (150 µl, 30.1 µl/min, 3 min). After that, the buffer-only control was performed by using 0.9% NaCl (150 µl, 0.75 µl/min, 3 min). An acceptable flow rate obtained in 0.9% NaCl before the start of analysis of patients' samples was established as less than 200 events/s. The total mean count rate of the buffer-only control was 49.8 events/s. The buffer-only control was analyzed at identical settings as for the samples of patients.

## 1.6 Buffer with reagents control

Buffer with reagents controls were performed identically as patients' samples, but 7.5 µl of 0.9% NaCl was added instead of the EV suspension. The total mean count rate of buffer with reagents controls were 159; 51; 90; and 110 events/s for antibody mixed with CD62P FITC, PAC-1 FITC, CD154 FITC, and CD31 FITC, respectively. The buffer with reagents control was analyzed at identical settings as for the samples of patients.

## 1.7 Unstained control

The unstained control was performed as a solution of EV suspension in 0.9% NaCl in the proportion 7.5 µl EV + 480 µl 0.9% NaCl. The unstained control was analyzed at identical settings as for the samples of patients. The total mean count rate of the unstained control was 354 events/s.

## 1.8 Trigger channel and threshold

Based on the buffer-only control, the trigger was established on the 488 nm laser, 488-MALS channel, at a threshold of 24 arbitrary units.

## 1.9 Flow rate quantification

The total events count in samples was enumerated by flow rate sensors of the flow cytometer. The correctness of counting was regularly checked by acquisition of calibration beads (Calibration Bead Mix, #1493, Apogee) with known particle concentrations (number per microliter), according to the product Performance Assessment supplied by Apogee. The ApogeeMix contains an aqueous mixture of spheres with diameters 180 nm, 240 nm, 300 nm, 590 nm, 880 nm, and 1300 nm with refractive index $\eta$=1.43 (silica, $SiO_2$), but also 110 nm and 500 nm green fluorescent beads with refractive index $\eta$=1.59 (polystyrene).


1. **Coumans FAW Brisson AR, Buzas EI, et al.** Methodological Guidelines to Study Extracellular Vesicles. *Circ Res.* 120, 2017, 1632-1648.

2. **Montoro-García S Shantsila E, Orenes-Piñero, Lozano ML, Lip G Y. H.** An innovative flow cytometric approach for small-size platelet microparticles: Influence of calcium. *Thromb Haemost.* 108:, 2012, 373–383.

**Table S1.** MIFlowCyt-EV checklist.

| Requirement | Requested Information |
|---|---|
| 1.1.Purpose | To compare the concentrations of extracellular vesicles (EVs) released from platelets ($CD61^+$), the procoagulant platelet-derived EVs ($CD61^+/PS^+$), subpopulations of platelet-derived EVs expressing CD62P ($CD61^+/CD62P^+$), active form of GPIIb/IIIa receptor ($CD61^+/PAC\text{-}1^+$), CD40L ($CD61^+/CD154^+$) and CD31 ($CD61^+/CD31^+$) in purified EV suspension made from PFP between patients after IS, healthy controls and patients burdened with risk factors of cardiovascular diseases and to compare the concentrations of pEV between different types of stroke. |
| 1.2.Keywords | Extracellular vesicles, flow cytometry, ischemic stroke, platelets, platelet reactivity |
| 1.3.Experiment variables | The study included 168 patients with ischemic stroke, 63 matched disease controls burdened with vascular disease risk factors (DC), and 21 healthy controls (HC). Total pEV concentration, defined as $CD61^+$ vesicles (pEV), the concentration of pEV with surface expression of phosphatidylserine ($PS^+$ pEV), the percentage of $PS^+$ pEV in the total pEV population (%$PS^+$ pEV) and the concentration of pEV with surface expression of proinflammatory (CD62P, CD40L, CD31) and procoagulant (PAC-1) markers were assessed on days 1 (D1), 3 (D3), 10 (D10) and 90 (D90) with the Apogee A50 Micro flow cytometer. Moreover, on D1, D3 and D10 after stroke, measurements of pEV microvesiculation after *ex vivo* platelet stimulation with the agonists ADP, TRAP, and arachidonic acid (AA) were performed and compared to the results obtained in HC. |
| 1.4.Organization name and address | Department of Neurology, Poznan University of Medical Sciences, Poznan, Poland<br><br>ul. Przybyszewskiego 49, 60-355 Poznań, Poland |
| 1.5.Primary contact name and email address | Joanna Maciejewska-Renkowska, jmaciejewska@ump.edu.pl |
| 1.6.Date or time period of experiment | January 2017 - May 2019; April 2019 - June 2020 |
| 1.7.Conclusions | The concentrations of pEV in the acute phase (D1 and D3) of stroke did not differ from those found in DC, while in the subacute (D10) and convalescent (D90) phases of stroke they were even lower. |

| | |
|---|---|
| | The percentage of $PS^+$ pEV in the studied populations was relatively low at 7-12%. The percentage of $PS^+$ pEV was greater in stroke subjects than in DC and HC. The percentage was the highest on D1 and then gradually diminished during recovery, although on D90 it was still higher than in controls.<br>All studied pEV parameters were higher in stroke subjects than in HC.<br><br>In the acute phase of stroke the vesiculation of pEV after *ex vivo* platelet stimulation with agonist is disturbed as compared to HC.<br><br>Thrombolytic treatment did not affect pEV concentration.<br><br>No significant differences in pEV concentrations depending on stroke etiology were observed. |
| 1.8. Quality control measures | Each day of work with flow cytometer started with cleaning the tube system with 7.5-10% NaClO solution at least once (150 µl, 30.1 µl/min, 3 min). After that, the buffer-only control was performed by using 0.9% NaCl (150 µl, 0.75 µl/min, 3 min). The buffer-only controls, buffer with reagents controls and unstained controls were prepared and analyzed at identical settings as for the samples of patients. The correctness of counting was regularly checked by acquisition of calibration beads (Calibration Bead Mix, #1493, Apogee, UK) with known particle concentrations (number per microliter), according to the product Performance Assessment supplied by Apogee Flow Systems. |
| 1.9. Other relevant experiment information | NA |
| 2.1.1.1. Sample description | Purified EV suspension made from PFP from human blood |
| 2.1.1.2. Biological sample source description | Blood samples were collected and processed following the guidelines of EV research. Citrated whole blood was centrifuged at 1500 g for 20 min at room temperature to obtain platelet-poor plasma (PPP). Obtained citrated plasma was carefully transferred to 1.5 ml Eppendorf 3810X tubes, pipetting the plasma 1 cm above the cellular pellet, and centrifuged at 13 000 *g* for 2 min to obtain platelet-free plasma (PFP). Then the prepared PFP (supernatant), pipetting the plasma 0.5 cm above the pellet, was aliquoted as 250 µl samples, and stored at −80 °C for further analysis.<br>Before EV isolation, PFP samples were thawed at room temperature. The first step of EV isolation included high-speed centrifugation of PFP for 30 min at 18 890 g. After centrifugation, 225 µl of supernatant was discarded |

| | |
|---|---|
| | and 225 µl of 0.22-µm-filtered PBS containing 1.4 mmol/l phosphate, 154 mmol/l NaCl, and 10.9 mM trisodium citrate with pH 7.4 was added to the pellet and then the second centrifugation was performed. After the second step, 225 µl of supernatant was discarded and 75 µl of PBS was added to the pellet, and the sample was briefly mixed on a vortex mixer. Immediately after, samples were put in liquid nitrogen for 20 min. After this stage, samples were ready to store at −80 °C. Finally, from a 250 µl PFP sample, 100 µl of isolated EV suspension was obtained. |
| 2.1.1.3. Biological sample source organism description | Hospitalized humans after ischemic stroke, healthy human controls and humans burdened with risk factors of vascular diseases. |
| 2.2 Sample characteristics | Purified EV suspension made from PFP. |
| 2.3. Sample treatment description | See section S 1.3., Table S3 |
| 2.4. Fluorescence reagent(s) description | See Table S2. |
| 3.1. Instrument manufacturer | Apogee, Hemel Hempstead, United Kingdom |
| 3.2. Instrument model | A50 Micro |
| 3.3. Instrument configuration and settings | All samples were measured for 3 minutes at a flow rate of 0.75 µl/min with sheath fluid pressure set at 150 mbar, on an A50-Micro flow cytometer equipped with two lasers: a 488 nm laser (50 mW), and 638 nm laser (75 mW). The peak height was measured for all channels. The PMT voltage for the 488-MALS detector was set at 300 V, with threshold set at 24 arbitrary units (a.u.). Signals from FITC were measured with a 488-Grn detector (FL1; 525/50 nm band pass filter, PMT voltage 550 V, threshold 21 a.u.), signals from PE were measured with a 488-Org detector (FL2; 575/30 nm band pass filter, PMT voltage 450 V, threshold 17 a.u.), and signals from APC were measured with a 638-Red detector (FL5; 680/35 nm band pass, PMT voltage 500 V, threshold 19 a.u.). After 3 minutes, the measurement was finished automatically, and then the flush procedure was automatically performed (5 cycles). After every two measurements, 7.5-10% NaClO solution was measured to additionally clean up the tube system (150 µl, 30.1 µl/min, 3 min) and then 0.9% NaCl was measured to ensure that no contamination from previous samples was present (150 µl, 0.75 µl/min, 3 min). |
| 4.1. List-mode data files | NA |
| 4.2. Compensation description | The fluorescence subtraction values were established by FMO control measurements. The values are listed in SM Figure S1. |
| 4.3. Data transformation details | No data transformations were applied. |

| | |
|---|---|
| 4.4.1. Gate description | The gates were established to isolate positive events in channels: 488-Grn, 488-Org, 638-Red. All of the antibodies applied in the protocol show the fluorescent signals from aggregates. Therefore, using buffer with reagents controls, we decided to recognize the fluorescence patterns of the antibodies and to set the gates in such a way as to omit the majority of positive events coming from antibodies aggregates. |
| 4.4.2. Gate statistics | The gate statistics included: number of positive events, number of positive events per microliter of sample, percentage of events counted in the gate compared to all counted events (ROI%). |

EV – extracellular vesicles; FMO – fluorescence minus one; IS – ischemic stroke; MALS – medium angle light scatter; NA – not applicable; PBS – phosphate-buffered saline; pEV – platelet-derived extracellular vesicles; PFP – platelet free plasma; PPP – platelet poor plasma; PS – phosphatidylserine; ROI – region of interests, gate; SM – supplementary materials

**Figure S2.** Fluorescence subtraction values used in experiment.

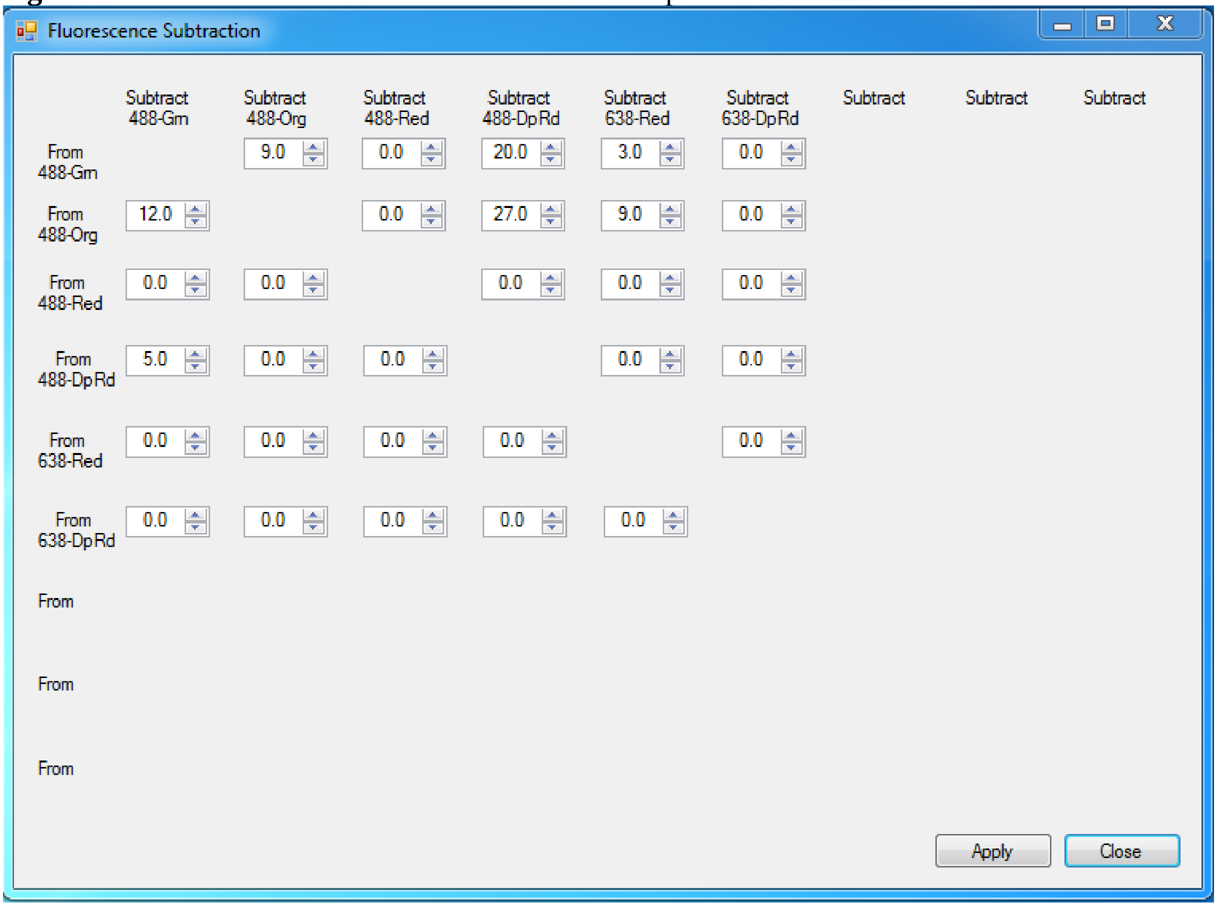

**Table S2.** Description of fluorescent reagents.

| Characteristic measured | Analyte | Analyte detector | Analyte reporter (fluorochrome) | Clone | Isotype | Reagent manufacturer name | Reagent catalogue number | Concentration of reagent during staining (µg/ml) |
|---|---|---|---|---|---|---|---|---|
| Integrin β3 | Human CD61 | Anti-CD61 antibody | PE | VI-PL2 | Mouse IgG1, κ | Becton Dickinson | 555754 | - |
| Phospholipid | Phosphatidylserine | Annexin V | APC | - | - | BioLegend | 640941 | 0.89 |
| Adhesion molecule | Human CD62P | Anti-CD62P antibody | FITC | AK4 | Mouse IgG1, κ | BioLegend | 304904 | 22.22 |
| Integrin | Active form of glycoprotein GPIIb/IIIa | Anti-PAC-1 antibody | FITC | PAC-1 | Mouse IgM, κ | BioLegend | 362804 | 11.11 |
| Inflammatory mediator | Human CD40L | Anti-CD154 antibody | FITC | 24-31 | Mouse IgG1, κ | BioLegend | 310804 | 22.22 |
| Adhesion molecule | Human platelet endothelial cell adhesion molecule (PECAM-1) | Anti-CD31 antibody | FITC | WM59 | Mouse IgG1, κ | BioLegend | 303104 | 22.22 |

APC – allophycocyanin; FITC – fluorescein isothiocyanate; PE – phycoerythrin; IgG – immunoglobulin G; IgM – immunoglobulin M.

**Table S3.** Description of reagents used in platelet stimulation tests.

| Platelet agonist | Platelet agonist full name | Reagent manufacturer | Reagent catalogue number | Concentration of reagent | Volume of reagent added to blood |
|---|---|---|---|---|---|
| TRAP | Thrombin receptor activator peptide (Ser-Phe-Leu-Leu-Arg-Asn-Pro-Asn-Asp-Lys-Tyr-Glu-Pro-Phe) | Sigma Aldrich | S-7152 | 0.5 mM | 30 µl |
| ADP | Adenosine 5′-diphosphate | Sigma Aldrich | A-6646 | 10 mM | 7.5 µl |
| AA | Arachidonic acid | Roche | 08847533190 (ASPItest) | 15 mM | 20 µl |
