# Supplementary material for: Prospective Quantitative and Phenotypic Analysis of Platelet-Derived Extracellular Vesicles and Its Clinical Relevance in Ischemic Stroke Patients
Source: Int J Mol Sci. 2024 Oct 18;25(20):11219. doi: 10.3390/ijms252011219 (PMC11508277; doi:10.3390/ijms252011219)
Supplement: Supplementary file 1 [file ijms-25-11219-s001.zip › SM3.pdf]

Supplementary Material S3.

Figure S3 A

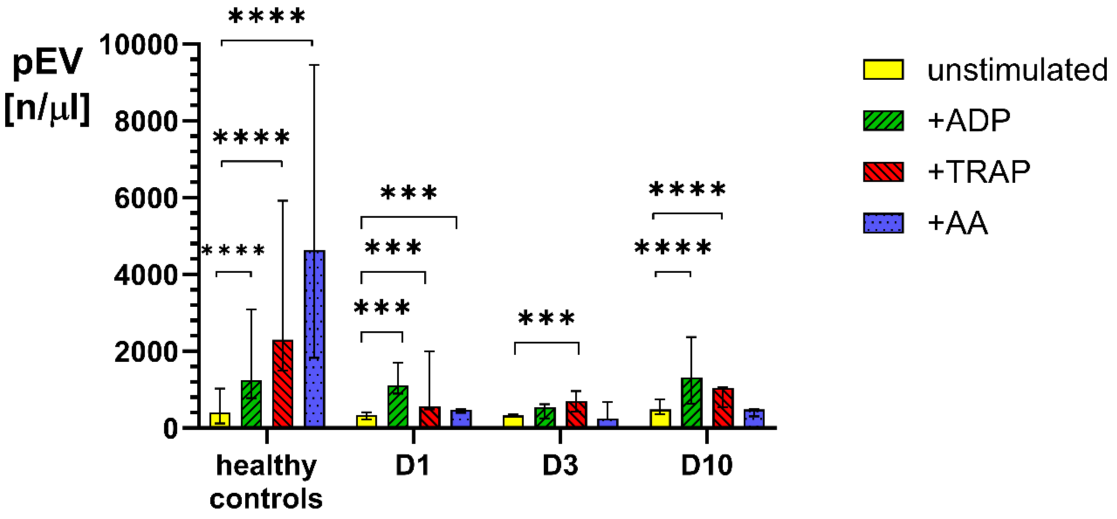

Figure S3 B

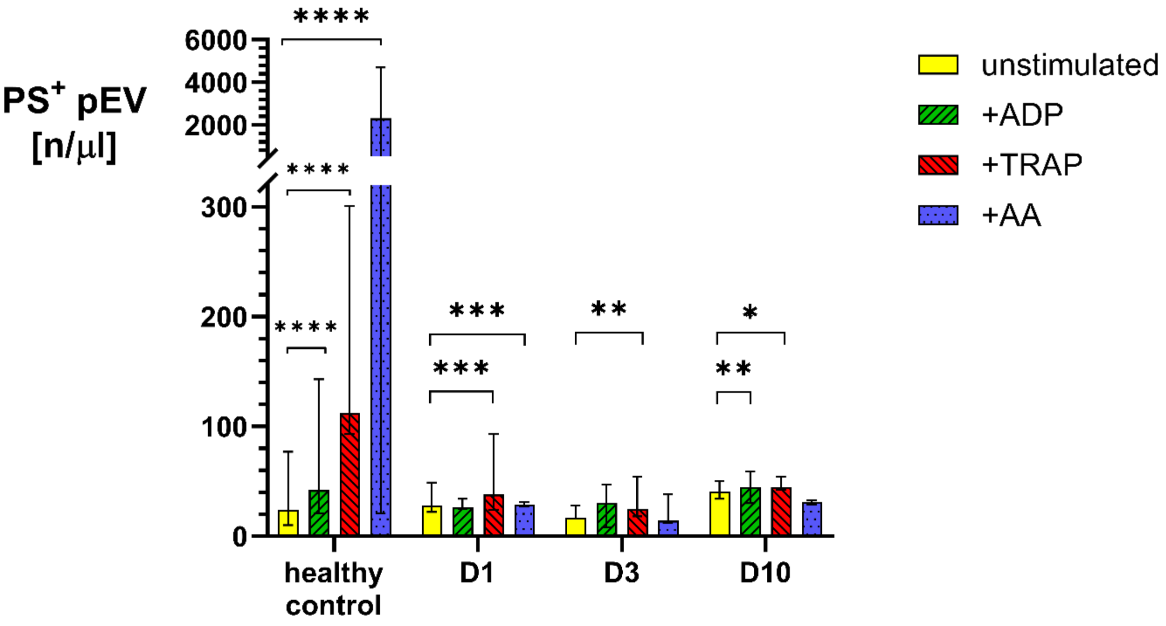

Figure S3 C

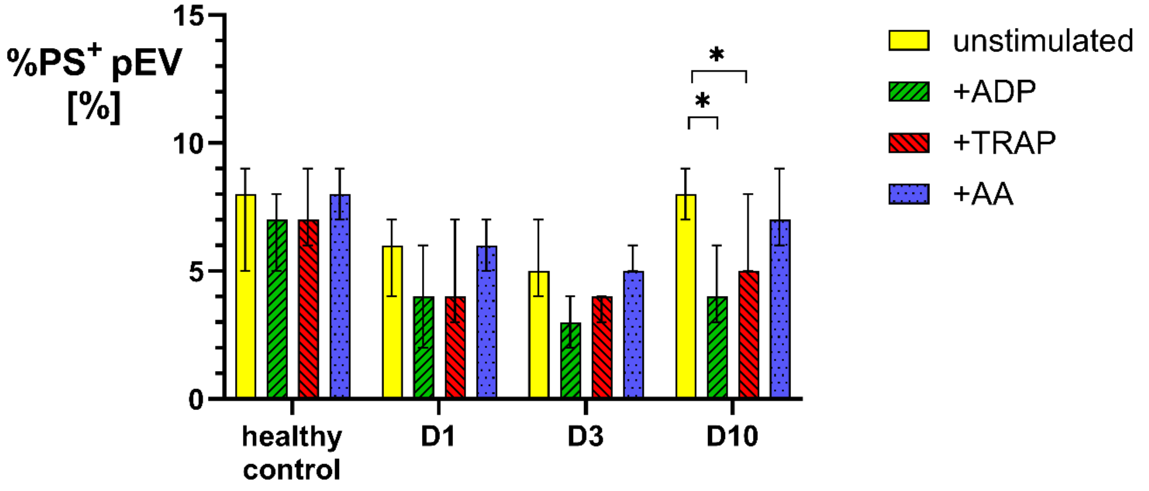

Figure S3 D

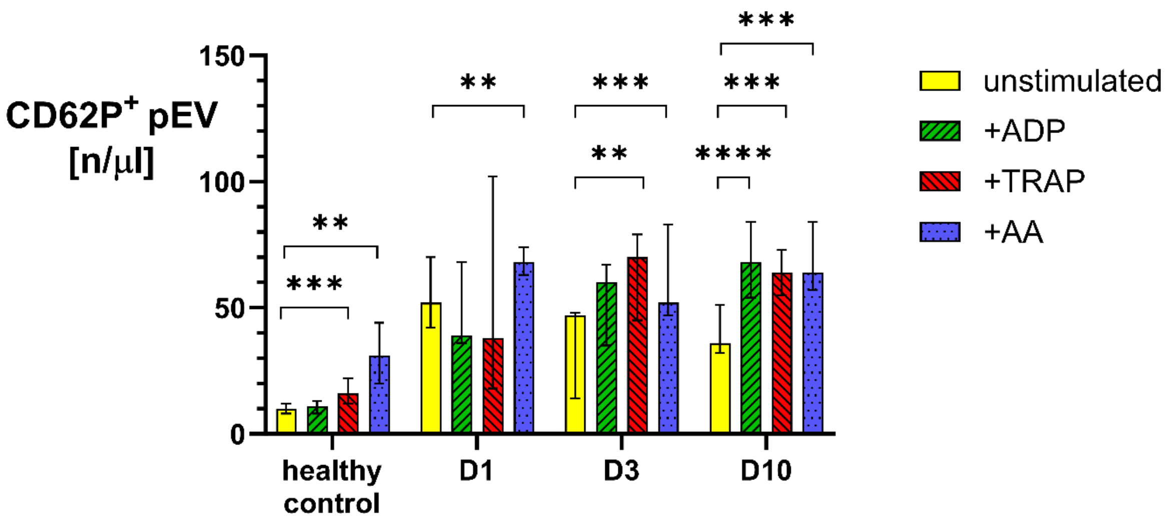

Figure S3 E

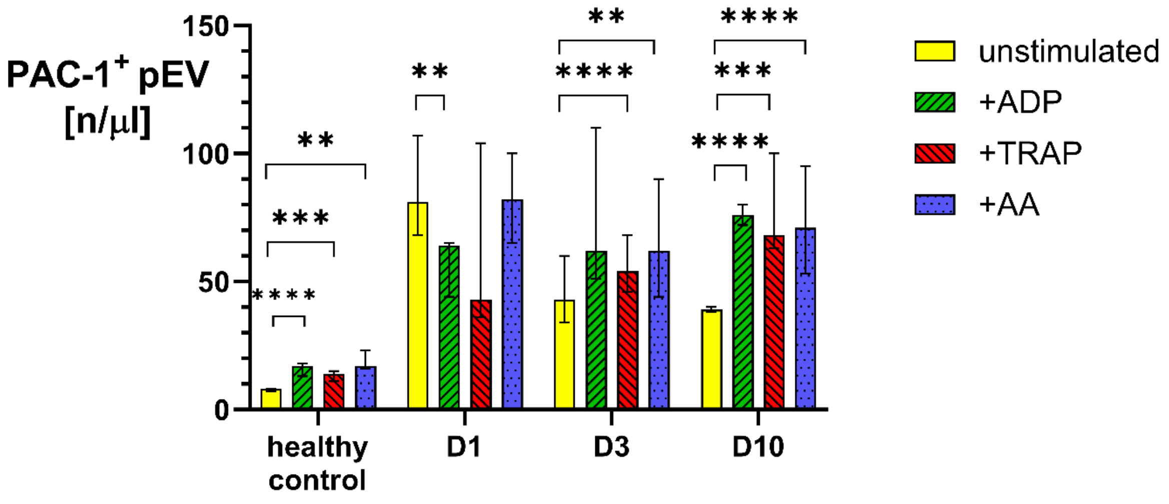

Figure S3 F

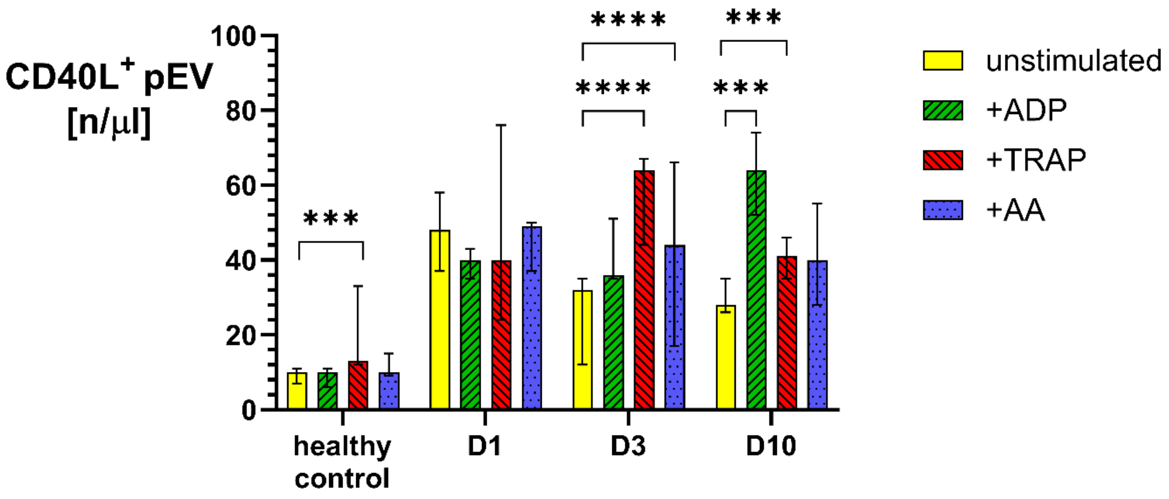

Figure S3 G

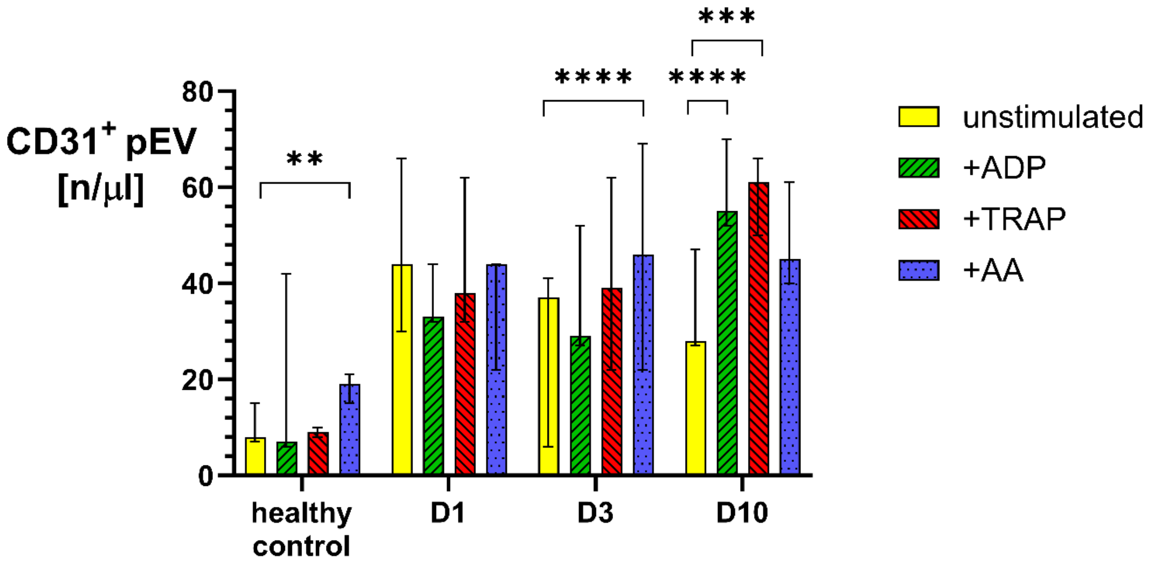

Figure S3. Concentrations of pEV (**A**) and PS+ pEV (**B**), percentage of PS+ pEV (**C**) and concentration of pEV with surface expression of CD62P (**D**), PAC-1 (**E**), CD40L (**F**), and CD31 (**G**) in patients with ischemic stroke (D1, D3, D10) and healthy controls before (unstimulated) and after *ex vivo* platelet stimulation with ADP, TRAP, and AA. **** $p<0.0001$; *** $p<0.001$; ** $p<0.01$; * $p<0.05$. Values are expressed as median (box) and IQR (whiskers).
