# Supplementary material for: Prospective Quantitative and Phenotypic Analysis of Platelet-Derived Extracellular Vesicles and Its Clinical Relevance in Ischemic Stroke Patients
Source: Int J Mol Sci. 2024 Oct 18;25(20):11219. doi: 10.3390/ijms252011219 (PMC11508277; doi:10.3390/ijms252011219)
Supplement: Supplementary file 1 [file ijms-25-11219-s001.zip › SM4.pdf]

Supplementary Material S4.

Table S4. Summary of treatment received by patients at admission and during the study.

D0<br>N=168			D1<br>N=168		D3<br>N=167				D10<br>N=163				D90<br>N=93	
**ASA**	**AC**	**r-tPA**	**ASA**	**AC**	**ASA**	**AC**	**LMWH**	**ASA + LMWH**	**ASA**	**AC**	**LMWH**	**ASA + LMWH**	**ASA**	**AC**
62	17	54	166	2	134	10	7	16	119	24	5	15	68	25

D0 – treatment at admission

D1 – day 1 – blood sampling within 24 hours since the stroke symptoms

D3, D10, D90 – day 3, day 10, day 90 of stroke

AC – anticoagulants

ASA – acetylsalicylic acid

r-tPA – recombinant tissue plasminogen activator

LMWH – low molecular weight heparin
