# Supplementary figures and images for: Prospective Quantitative and Phenotypic Analysis of Platelet-Derived Extracellular Vesicles and Its Clinical Relevance in Ischemic Stroke Patients
Source: Int J Mol Sci. 2024 Oct 18;25(20):11219. doi: 10.3390/ijms252011219 (PMC11508277; doi:10.3390/ijms252011219)

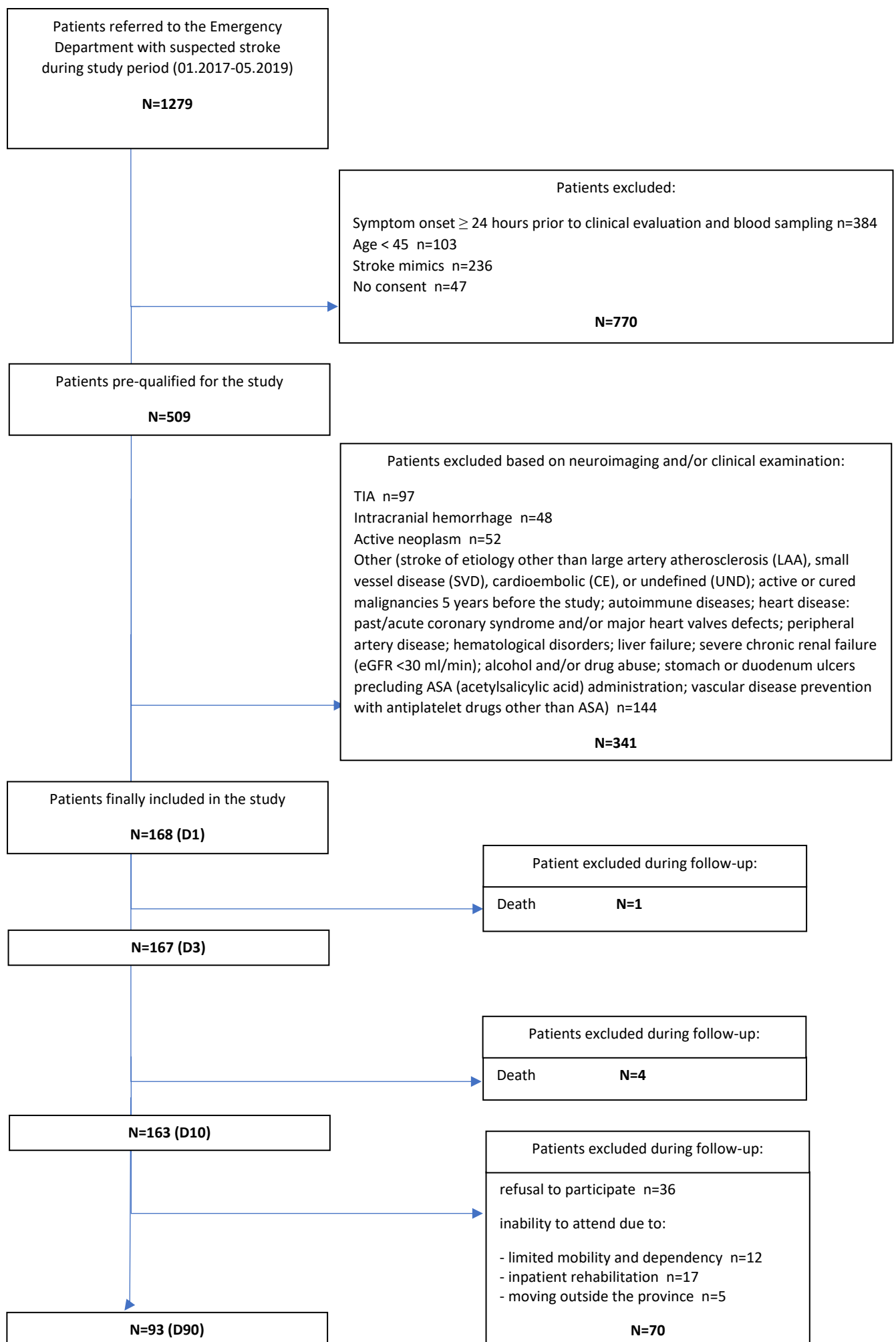

**Supplementary Material S1.**

Figure S1. Flow chart of participant recruitment

Supplement: Supplementary file 1 [file ijms-25-11219-s001.zip › SM1.pdf]
